# Foreword to the special virtual issue on *Actinide physics and chemistry with synchrotron radiation*

**DOI:** 10.1107/S1600577522007019

**Published:** 2022-08-10

**Authors:** Kristina O. Kvashnina, Sergei M. Butorin, Shuao Wang, Weiqun Shi

**Affiliations:** a The Rossendorf Beamline at ESRF, The European Synchrotron, CS40220, 38043 Grenoble Cedex 9, France; b Institute of Resource Ecology, Helmholtz Zentrum Dresden-Rossendorf (HZDR), PO Box 510119, 01314 Dresden, Germany; c Condensed Matter Physics of Energy Materials, X-ray Photon Science, Department of Physics and Astronomy, Uppsala University, PO Box 516, SE-75120 Uppsala, Sweden; d State Key Laboratory of Radiation Medicine and Protection, School for Radiological and Interdisciplinary Sciences (RAD-X) and Collaborative Innovation Center of Radiation Medicine of Jiangsu Higher Education Institutions, Soochow University, Suzhou 215123, People’s Republic of China; e Laboratory of Nuclear Energy Chemistry, Institute of High Energy Physics, Chinese Academy of Sciences, Beijing 100049, People’s Republic of China

**Keywords:** actinides, synchrotron radiation

## Abstract

Foreword to the virtual special issue of *Journal of Synchrotron Radiation* on *Actinide Physics and Chemistry with Synchrotron Radiation*.

Actinide research is currently experiencing a renaissance in the fields of material science, nanotechnology, medicine and environmental science. It is now possible to study the chemistry and physics of the actinide elements (all radioactive) using state-of-the-art non-destructive techniques at synchrotrons which have not been available before. The beamlines and instruments dedicated to actinide research have made various spectroscopic and scattering methods accessible to scientists worldwide. The new synchrotron sources at the large-scale facilities offer more advanced possibilities for the development of new methodologies in actinide science in the future. Theoretical studies of actinides are followed by unique experimental methods and novel experimental data.

The virtual special issue on *Actinide Physics and Chemistry with Synchrotron Radiation* (https://journals.iucr.org/special_issues/2022/actphyschem/index.html) includes several invited contributions that focus on novel results obtained for actinide materials with the help of synchrotron radiation. In total, 19 articles cover a broad variety of synchrotron-based spectroscopic and scattering methods used to study actinide-containing materials and can be tentatively divided into five groups:

(1) Gerry Lander and Roberto Caciuffo reviewed the X-ray diffraction (XRD), resonant X-ray scattering (RXS), X-ray magnetic circular dichroism (XMCD), resonant and non-resonant inelastic scattering (RIXS, NIXS), and dispersive inelastic scattering (IXS) experiments in studies of actinide materials (Caciuffo & Lander, 2021 ▸). Advanced synchrotron-based spectroscopic methods, such as X-ray absorption near-edge structure (XANES), recorded in the high energy resolution fluorescence detection (HERFD) mode at the U *L*_3_- and *M*_4_-edges were used by Sergei Butorin and co-workers in the chemical state investigation of uranium carbides supported by single Anderson impurity model (SIAM) theory (Butorin *et al.*, 2022 ▸). Rene Bes and co-authors reported HERFD-XANES data at the U *L*_1_-edge of KUO_3_ analysed by electronic structure calculations (Bes *et al.*, 2022 ▸). Tim Pruessmann and co-workers applied the HERFD/RIXS methodology at lanthanide and actinide *L*_3_-edges in combination with low-*Z* element *K*-edge XANES along with electronic structure calculations to probe the chemical and physical properties of *f*-electron systems (Pruessmann *et al.*, 2022 ▸).

(2) Alexander Scott Ditter and co-authors showed the power of soft X-ray spectromicroscopy at the O *K*-, U *N*_4,5_- and Ce *M*_4,5_-edges for spent nuclear fuel investigations, yielding chemical information on the sub-micrometre scale (Ditter *et al.*, 2022 ▸). Yusheng Zhang and co-workers investigated the covalency effects in the bonding between the uranyl ion and di­thio­phosphinate by combining sulfur *K*-edge XANES and density functional theory (Zhang *et al.*, 2022 ▸).

(3) Lin Wang and co-authors combined X-ray diffraction and extended X-ray absorption fine-structure (EXAFS) methods to study thorium(IV) adsorption onto multi-layered titanium carbides, Ti_3_C_2_T_x_ (Wang *et al.*, 2021 ▸). Jian Sun and co-authors studied two series of uranium-doped Nd_2_Zr_2_O_7_ pyrochlore materials as potential nuclear waste host matrices by the combination of the X-ray diffraction, Raman and EXAFS techniques (Sun *et al.*, 2022 ▸). Using EXAFS at the U *L*_3_-edge and XRD, Antonia S. Yorkshire and co-authors investigated U(VI)–cement mineral interactions, relevant to understanding the waste disposal of actinide-containing materials (Yorkshire *et al.*, 2022 ▸). Hao Ding and co-workers examined synthetic Chernobyl lava specimens using micro-focus spectroscopy at the U *L*_3_-edge along with diffraction techniques and were able to construct U oxidation state maps (Ding *et al.*, 2021 ▸). Anna Krot and co-workers performed U(VI) speciation in contaminated environments using EXAFS data recorded at the U *L*_3_-edge on U reference compounds (Krot *et al.*, 2022 ▸). Cyril Zurita and co-workers utilized Pu *L*_3_ EXAFS to study the interaction of Th(IV), Pu(IV) and Fe(III) with ferritin protein (Zurita *et al.*, 2022 ▸).

(4) Thomas Dumas and co-authors studied the size and structure of the hexanuclear plutonium oxo-hydroxo clusters in an aqueous solution by combining small-angle X-ray scattering (SAXS) and Pu *L*_3_-edge EXAFS methods (Dumas *et al.*, 2022 ▸). Anna Romanchuk and co-workers proposed a new core-shell approach for the actinide and lanthanide dioxide nanoparticles with calculated effective coordination numbers from Ce *K*-edge and Th and Pu *L*­_3_-edge EXAFS (Romanchuk *et al.*, 2022 ▸). Baihui Zhai and co-authors studied the formation and structure of polynuclear thorium(IV) colloids and thorium dioxide nanoparticles by SAXS (Zhai *et al.*, 2022 ▸).

(5) Korey P. Carter and co-authors reported on the *in situ* beam reduction of Pu(IV) and Bk(IV) during Pu/Bk *L*_3_ XANES/EXAFS measurements, which yielded Pu(III) and Bk(III) coordination complexes with hy­droxy­pyridinone chelators (Carter *et al.*, 2022 ▸). Richard Husar and co-workers performed *in situ* Np *L*_3_-edge XANES/EXAFS measurements and density functional theory calculations in combination with an electrochemical setup, dedicated to radioactive samples (Husar *et al.*, 2022 ▸). Damien Prieur and co-workers conducted *in situ* U *L*_3_-edge XANES measurements and thermodynamic calculations to establish the U–O phase diagram (Prieur *et al.*, 2021 ▸). Bianca Schacherl and co-authors implemented the cryogenic sample environment for the tender X-ray range, necessary for actinide HERFD *M*_4,5_-edges data collection on radioactive samples (Schacherl *et al.*, 2022 ▸).

There is no doubt that synchrotron radiation plays a critical role in understanding the physics and chemistry of actinide-containing materials. We very much look forward to continued developments in experimental and theoretical synchrotron-based methodologies for fundamental and applied actinide science in the future.
